# The Oncogenic Role of ARG1 in Progression and Metastasis of Hepatocellular Carcinoma

**DOI:** 10.1155/2018/2109865

**Published:** 2018-09-18

**Authors:** Jia You, Wei Chen, Jing Chen, Qi Zheng, Jing Dong, Yueyong Zhu

**Affiliations:** Liver Research Center, First Affiliated Hospital of Fujian Medical University, Fuzhou 350005, Fujian Province, China

## Abstract

ARG1, which encodes Arginase1, is expressed in the liver cytoplasm and plays a major role in the hepatic urea cycle. The past research works shed light on the fact that ARG1 participates in anti-inflammation, tumor immunity, and immunosuppression-related diseases. Nevertheless, the concrete role and clinical significance of ARG1 in the progression of hepatocellular carcinoma (HCC) remain unclear. Herein, we aimed at examining the expression and clinicopathological significance of ARG1 in HCC, together with determining the effect of ARG1 on the progression and metastasis of HCC. In the current study, evaluation of the expression of ARG1 and clinicopathological significance of ARG1 was carried out in the human HCC tissues microarray, and the ARG1 overexpression vector and shRNA-ARG1 plasmids were constructed for the assessment of the concrete effect of ARG1 on cellular behaviors of Huh7 cells. As our data revealed, ARG1 was significantly downregulated in HCC, and the higher expression of ARG1 was positively correlated with more aggressive tumor growth, size, ALT, and GGT level. Significantly, we found that the high expression of ARG1 was correlated with poor DFS of HCC patients. Besides, in vitro study revealed that overexpression of ARG1 could enhance arginase activity, cell viability, migration, and invasion of Huh7 cells, and loss-of-function of ARG1 by shRNA interference could inhibit these cellular behaviors. Additionally, overexpression of ARG1 led to a significant increase in the expression of Vimentin, N-cadherin, and *β*-catenin both at protein and mRNA levels, which promotes the EMT process. On the other hand, these proteins' expression was significantly downregulated in ARG1 silenced Huh7 cells. Besides, the level of E-cadherin protein was upregulated in ARG1 knocked down cells. In conclusion, ARG1 might play a pivotal role as an oncogene in the progression of HCC through promoting the EMT process.

## 1. Introduction

Hepatocellular carcinoma (HCC) is termed as a common and aggressive malignancy, which ranks as the second leading cause of the cancer-related deaths, annually leading to more than 110,000 mortalities worldwide [1–3]. Despite recent advances in our understanding of the molecular basis as well as treatment of HCC, the 5-year survival rate of HCC is still under 5% [4]. Poor prognosis forces us to explore new therapeutic strategies for HCC. The tumorigenesis and development of HCC are multistep process, involving an array of gene expression profiles and intracellular signaling pathway dysregulation [5]. Therefore, it is quite critical to identify relevant genes and novel targets for therapy of HCC.

Arginase is considered as a pivotal metabolic enzyme, which catalyzes the hydrolysis of arginine to ornithine and urea. Since the L-ornithine produced by arginase will further metabolize to polyamines, which is involved in the multiple fundamental cellular functions, arginase has been revealed to impact an array of pivotal downstream metabolic pathways [6]. In mammals, arginase features two distinct isoforms, among which arginase I (encoded by* ARG1*) is expressed in the liver cytoplasm, as well as substantially contributing to the hepatic urea cycle [6, 7]. Arginase II (encoded by* ARG2*) is a mitochondrial enzyme with a wide range of tissue distribution, mainly expressed in the kidney, brain, small intestine, prostate, breast, and macrophages [8]. Recent studies confirm that ARG1 is induced in alternatively activated (M2) macrophages and participates in anti-inflammation, tumor immunity, tumor proliferation, metastasis, and immunosuppression-related diseases [9, 10]. Based on these researches, ARG1 might be a potential target for cancer therapy. Nevertheless, the clear effect and clinicopathological significance of ARG1 on the HCC progression continue being unclear.

In the present study, we aimed to examine the expression and clinicopathological significance of ARG1 in HCC and, furthermore, figure out the role of ARG1 in the progression and metastasis of HCC. Our data reported that ARG1 was downregulated in HCC tissues and was correlated with prognosis of patients. We also identified that ARG1 functioned as an oncogene in HCC, based on the fact that knockdown of ARG1 by shARG1 interference could decrease cell proliferation activity and motility of HCC cell, while ARG1 overexpression could promote tumor-related phenotypes in HCC cells. As the results indicate, ARG1 might be a potential prognosis predictor for HCC patients, together with being a novel target for HCC treatment.

## 2. Materials and Methods

### 2.1. Tissue Samples

The human HCC tissues microarray was obtained from Shanghai Outdo Biotech Company (LivH180Su06, Shanghai, China), which included HCC tissues and corresponding paracancerous tissues from 90 cases of HCC patients. The clinicopathological information of the HCC patients, including age, gender, tumor size, tumor envelope, relapse, cirrhotic nodule, pathological grading, total bilirubin (TB), alanine aminotransferase (ALT), glutamyltransferase (GGT), and alpha fetoprotein (AFP) levels, was summarized in Table 2.

### 2.2. Cell Culture and Transfection

The HCC cell line Huh7 was obtained from the Cell Bank of the Chinese Academy of Sciences (Shanghai, China), followed by culturing in DMEM (Dulbecco's Modified Eagle Medium, Thermo Fisher, USA) medium containing 10% FBS (Gibco, USA) at 37°C under 5% CO_2_. Huh7 cells were infected with lentiviral particles loading the overexpression ARG1 plasmid (OE-ARG1), overexpression control plasmid (OE-NC), shRNA-ARG1 plasmid (sh-ARG1), and shRNA control plasmid (sh-NC), correspondingly. And Huh7 cells without any treatment were used as the control group (CON).

### 2.3. Plasmid Constructions and Lentiviral Constructs

The ARG1 overexpression plasmid, shRNA-ARG1 plasmids, and their negative control plasmids were packaged into lentiviral particles. The full CDS sequence of ARG1 was amplified and cloned into pLenO-GTP (Biotheon Technologyco., LTD, Fuzhou, China). Three shRNA sequences targeting ARG1 were synthesized, and the sequences were as follows: shRNA-ARG1-1#: GCAGCAAAGAGAAGTGTCAGA; shRNA-ARG1-2#: GGATTATTGGAGCTCCTTTCT; shRNA-ARG1-3#: GCCCTACAGTATTGAGAAAGG. The pLenO-gph (Biotheon Technologyco) vector was performed to construct shRNA plasmid. Tronolab system (Tronolab, Switzerland) was adopted for lentivirus packaging to obtain stably expressing ARG1, shRNA-ARG1, or negative control (NC), which were used to infect Huh7 cells.

### 2.4. Immunohistochemistry Assay

EliVisionTMplus kit (Maixin, China) was used for immunohistochemistry assay in accordance with the manufacturer's instructions [11]. The anti-ARG1 was purchased from Abcam (USA). Determination of the ARG1 immunostaining score was made by the sum of staining intensity and positive stained cells rate. The staining intensity was graded as follows: no staining (0); weak staining (1); moderate staining (2); and strong staining (3). The positive stained cells rate was graded as follows: 0 ~ 5% (0); 5% ~ 25% (1); 26% ~ 50% (2); 51% ~ 75% (3); and >75% (4). The final score was the sum of the two sets of scores, and the score lower than 2 was regarded as negative staining, ≤ 3 as ARG1 low expression, and >3 as ARG1 high expression.

### 2.5. RT-PCR

Total RNA was extracted using Trizol (PuFei, China) and then reverse transcribed to cDNA using SuperRT cDNA Synthesis kit (CWBIO, Beijing, China). The expression of gene mRNA was examined using SYBR Master Mixture (Takara, Ohtsu, Japan). Primers were as follows: ARG1: Upstream: 5′- TTGGCTTGAGAGACGTGGAC -3′, Downstream: 5′- GTGCCAGTAGCTGGTGTGAA -3′; Vimentin: Upstream: 5′- GACGCCATCAACACCGAGTT-3′, Downstream: 5′- CTTTGTCGTTGGTTAGCTGGT-3′; N-cadherin: Upstream: 5′- AGCCAACCTTAACTGAGGAGT-3′, Downstream: 5′- GGCAAGTTGATTGGAGGGATG-3′; *β*-catenin: Upstream: 5′- ATGGCTTGGAATGAGAC-3′, Downstream: 5′- AACTGGATAGTCAGCACC-3′; *β*-actin: Upstream: 5′- ACTCGTCATACTCCTGC -3′, Downstream: 5′- GAAACTACCTTCAACTCC -3′. The comparative Ct (ΔΔct) method was used to analyze the obtained RT-PCR data.

### 2.6. Western Blot

Protein was extracted by using RIPA Lysis Buffer (CWBIO), followed by being quantified using a BCA Protein Assay Kit (Beyotime, Shanghai, China). Protein (20 *μ*g) of every sample was separated using 10% SDS-PAGE gel, followed by transferring onto the PVDF membrane. Thereafter, the PVDF membrane was blocked in 5% nonfat milk and incubated with the primary antibodies at 4°C overnight. Subsequently, incubation of the membrane with the secondary antibodies was carried out for 1 h, followed by incubation with the ECL substrate for the signal development. ARG1, Vimentin, N-cadherin, *β*-catenin, and *β*-Actin antibodies were obtained from Abcam (Cambridge, UK); besides that, the secondary antibodies were obtained from Proteintech Group (IL, USA).

### 2.7. Determination of Arginase Activity

Almost 1×10^4^ transfected cells were harvested and lysed in Tris-HCl containing 1*μ*M pepstatin A, 1*μ*M leupeptin, and 0.4% (W/V) Triton X-100, followed by centrifugation for 10 min to get cell lysate. The arginase activity was examined using an Arginase Activity Assay Kit (Sigma-Aldrich, Missouri, USA) according to the instructions.

### 2.8. Cell Viability Assay

CCK8 assay was carried out for the examination of cell viability. About 2×10^4^ infected Huh7 cells were seeded into each well of 96-well plates and cultured for 48 h. The cells were further cultured following the addition of CCK8 reagent (10 *μ*l, Solarbio Science & Technology, Beijing, China) into each well. After 3h, the OD value of excitation light was measured. Cell viability was defined and calculated by the following formula: [OD (experimental group)-OD (blank)]/ [OD (CON group) -OD (blank)].

### 2.9. Cell Invasion and Migration Assay

Cell wound scratch assay and Transwell assay were performed to examine cell migration. With regard to cell wound scratch assay, about 2×10^4^ infected Huh7 cells per well were seeded into both sides of the scratch plate (NEST, Wuxi, China) for 24 h incubation, followed by taking out the separator and taking pictures under the microscope (Olympus, Japan). Subsequent to a period of 24 h, the cell migration was observed under the microscope and pictures were taken. With regard to Transwell migration assay, about 4×10^4^ infected Huh7 cells were cultured with medium in the upper chamber (Millipore, MA, USA) for 24 h. Subsequent to that, cells were fixed with methanol for 30 min. Thereafter, the filters were stained with 0.1% crystal violet for 25 min, followed by observing under the microscope and taking pictures. With regard to Transwell invasion assay: the Transwell chambers were coated with Matrigel (BD Bioscience, New Jersey, USA). About 4×10^4^ infected Huh7 cells were added to the top chamber, and medium with 20% FBS was added to the lower chamber. Subsequent to a period of 24 h, invaded cells were stained using 0.1% crystal violet for 25 min, and photographed under the microscope.

### 2.10. ELISA Assay

After being infected for 20 h, cells were centrifuged at 2,000 × g for 10 min, and cell culture supernatants were collected to assay using the Human E-cadherin SimpleStep ELISA kit (Abcam) and Human Arginase 2 (ARG2) ELISA Kit (KALANG, Shanghai, China) according to the instructions.

### 2.11. Statistical Analysis

In the current study, the data were presented as the mean ± SE, and the statistical analysis was carried out with the use of the SPSS 20.0. The Pearson Chi-Square analysis was employed to analyze the correlation between expression of ARG1 and clinicopathological characteristics of HCC patients. Student's* t* test was used to analyze the differences between two groups, and one-way ANOVA was performed to compare three or more groups. P<0.05 was considered significant.

## 3. Results

### 3.1. Expression of ARG1 Is Downregulated in HCC and Significantly Correlated with Patients' Prognosis

**Figure 1:**
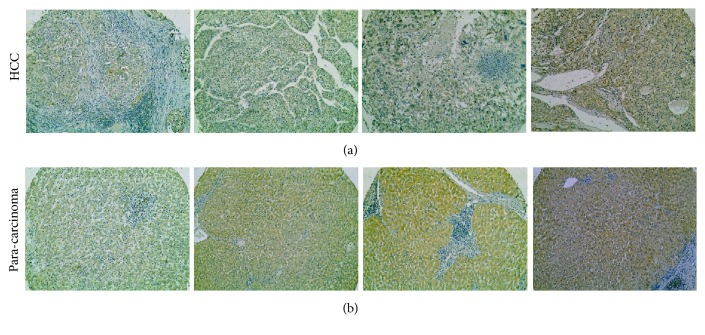
ARG1 is downregulated in HCC. Immunohistochemical staining of ARG1 in HCC tissues ((a)×100) and paracarcinoma ((b)×100). These 4 samples were from 90 cases in the human HCC tissues microarray.

To determine the expression of ARG1 in HCC, the HCC tissues microarray was carried out. As evident from Figure 1, the ARG1 positive signaling primarily located in the cytoplasm of tumor cells and paracancerous cells. Dramatically downregulated expression of ARG1 in HCC tissues (81.1%, 73/90) was observed, in comparison with that in corresponding paracarcinoma tissues (13.3%, 12/90, P=0.001, Table 1). Besides that, it was observed that the expression level of ARG1 was closely related to several clinicopathological features of HCC (Table 2). The expression of ARG1 in small tumors (diameter< 5cm, 9.1%, 4/44) was significantly lower as compared with that in the large tumors (diameter≥ 5cm, 28.3%, 13/46, P=0.020), suggesting that the expression of ARG1 might be associated with tumor growth. The low expression rate of ARG1 in the pathological grades I-II (87.9%, 51/58) was significantly higher as compared with that in the pathological grades III-IV (68.8%, 22/32, P=0.026, Table 2). In addition, the expression of ARG1 had significant correlation with both the ALT level (alanine aminotransferase, P=0.010) and GGT level (glutamyltransferase, P=0.014). Importantly, we figured out that the expression of ARG1 in the patients with well disease-free survival (DFS, ≥12, 13.2%, 9/68) was significantly lower as compared with that in the patients with poor DFS (<12, 38.1%, 8/21, P=0.027). The results presented above indicated that the expression of ARG1 might be associated with the progression of HCC and have prognostic value that higher expression of ARG1 is correlated with poor prognosis.

### 3.2. ARG1 Enhances Cell Viability of Huh7 Cells

**Figure 2:**
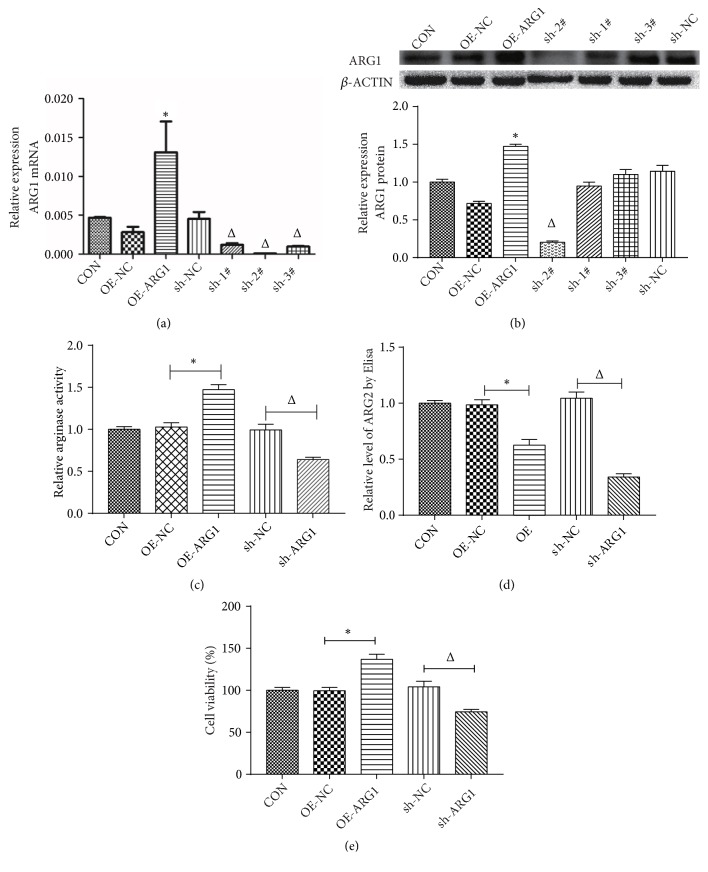
ARG1 promotes cell proliferation of Huh7 cells. ((a) and (b)) The expression of ARG1 was significantly upregulated in overexpression cell group (OE-ARG1) and inhibited in shRNA-ARG1-2# interference cell group (sh-2#) both at mRNA (a) and protein (b) level. Then, shRNA-ARG1-2# was used in the further experiment. *β*-Actin mRNA was performed as a control in RT-PCR assay, *β*-actin was used as a loading control in Western blot assays, and the relative expression level of ARG1 protein was normalized to that in CON group. (c) Determination of arginase activity in ARG1 overexpression cells and knockdown cells. (d) ELISA assay suggests that upregulation of ARG1 reduces the protein level of ARG2 in Huh7 cells, and downregulation of ARG1 also reduces its expression. (e) CCK8 assay showed that overexpression of ARG1 increased cell viability of Huh7 cells, and ARG1 knockdown inhibited cell viability. N=3, ^*∗*^P<0.05 compared to the OE-NC; ^Δ^P<0.05 compared to the sh-NC.

ARG1 was observed to be dysregulated in HCC. Besides that, the effects of ARG1 on biological behaviors of HCC cells were further examined as well. Accordingly, the HCC cell Huh7 was transfected with pLenO-ARG1 or shARG1 to obtain stable ARG1 overexpressed cells (OE-ARG1) and ARG1 silenced cells (shARG1), respectively; pLenO (OE-NC) and shRNA control (sh-NC) were used as the negative control, correspondingly. As shown in Figure 2(a), the expression of ARG1 mRNA was clearly upregulated in OE-ARG1 group as compared with the OE-NC (P<0.05); and sh-ARG1-2# significantly blocked the expression of ARG1 mRNA in comparison with the sh-NC group (P<0.05). Thereafter, Western blot assay was performed to further verify the efficiency of overexpression or knockdown of ARG1 in Huh7 cells. The results showed that the expression of ARG1 was also significantly upregulated in OE-ARH1 group (P<0.05) and decreased by shARG1-2# (P<0.05) at protein level (Figure 2(b)). Furthermore, the arginase activity in Huh7 cells was also significantly increased by ARG1 overexpression, while it decreased by sh-ARG1 interference (Figure 2(c)). Interestingly, we also observed that the expression level of arginase II (ARG2) was also affected by ARG1 in Huh7 cells, which was decreased in both ARG1 overexpressed and silenced cells (Figure 2(d)).

CCK8 assay was employed for the detection of the cell proliferation activity and viability of Huh7 cells, highlighting that the ARG1 overexpression could significantly improve the cell viability of Huh7 cells (P<0.05, Figure 2(e)). In the meantime, ARG1 knockdown significantly reduced the cell viability of Huh7 cells (P<0.05, Figure 2(e)), further confirming that ARG1 promotes cell viability of HCC cells in vitro.

### 3.3. ARG1 Promotes Cell Motility of Huh7 Cells

**Figure 3:**
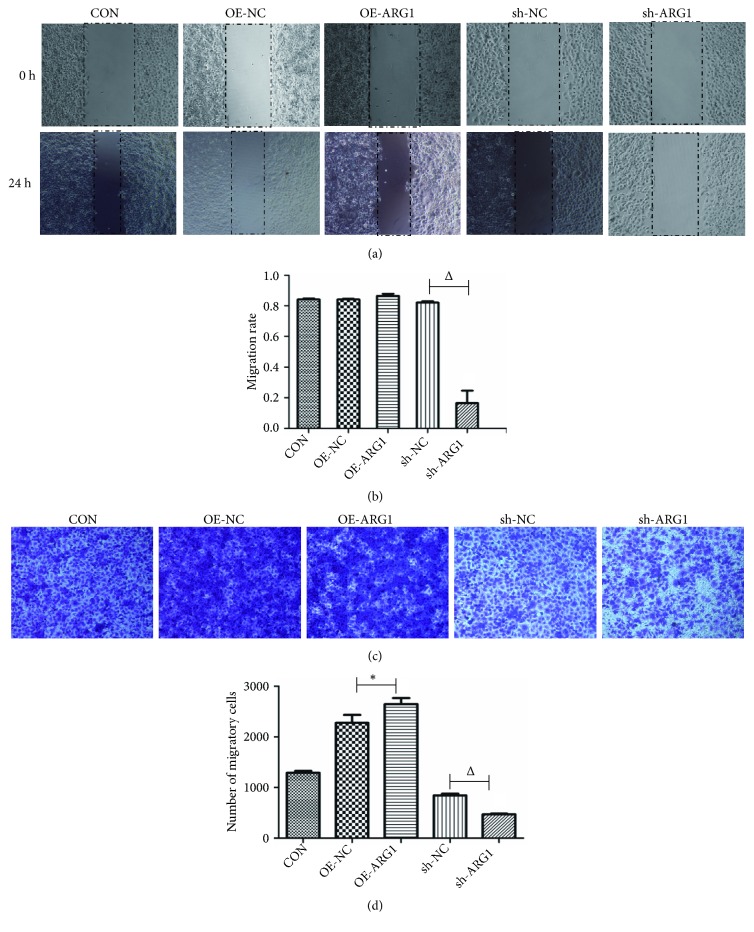
ARG1 promotes cell migration of Huh7 cell. ((a) and (b)) Cell wound scratch assay showed that ARG1 knockdown significantly suppressed cell migration rate of Huh7 cells, while overexpression of ARG1 had no significant effect on cell migration. ((c) and (d)) Transwell assay showed that overexpression of ARG1 promoted cell migration activity of Huh7 cells, and ARG1 knockdown inhibited cell migration activity. N=3, ^*∗*^P<0.05 compared to the OE-NC; ^Δ^P<0.05 compared to the sh-NC.

**Figure 4:**
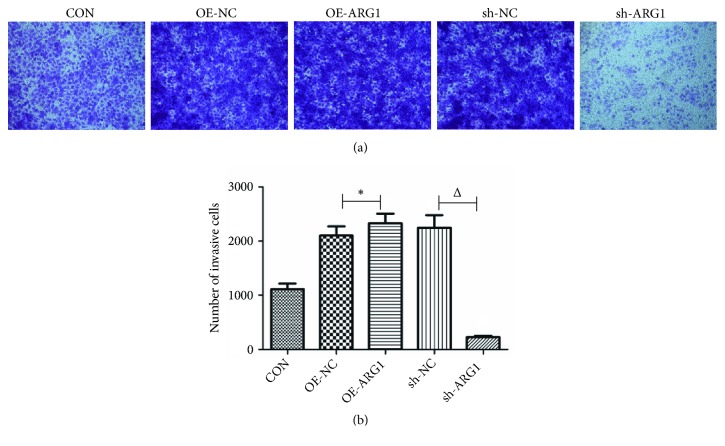
ARG1 promotes cell invasion of Huh7 cell. ((a) and (b)) Transwell assay showed that overexpression of ARG1 promoted cell invasion of Huh7 cells, and ARG1 knockdown inhibited cell invasion. N=3, ^*∗*^P<0.05 compared to the OE-NC; ^Δ^P<0.05 compared to the sh-NC.

To investigate the effect of ARG1 on tumor metastases, cell migration and invasion were examined using cell wound scratch assay and Transwell assays, correspondingly. As shown in Figures 3(a) and 3(b), the cell wound scratch assay revealed that ARG1 knockdown markedly reduced cell migration ability of Huh7 cells in comparison with the sh-NC group (P<0.05), while overexpression of ARG1 did no significantly impact cell migration of Huh7 cells (P>0.05). The inhibition effect of ARG1 knockdown on cell migration was further substantiated by a Transwell assay (P<0.05, Figures 3(c) and 3(d)). Together with that, the Transwell assay also revealed that overexpression of ARG1 could significantly increase cell migration ability of Huh7 cells (P<0.05, Figures 3(c) and 3(d)). Moreover, overexpression of ARG1 also enhanced cell invasion ability of Huh7 cells, and loss of ARG1 by shARG1 interference accordingly reduced cell invasion ability of Huh7 cells (both P<0.05, Figures 4(a) and 4(b)). Taken together, it was concluded that ARG1 could promote the cell motility of HCC cells in vitro.

### 3.4. ARG1 Promotes the Epithelial-to-Mesenchymal Transition (EMT) in Huh7 Cells

**Figure 5:**
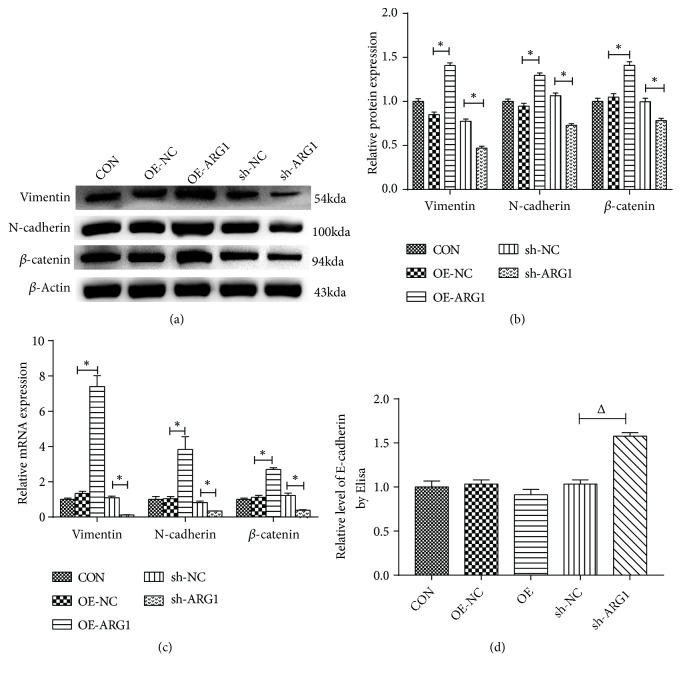
ARG1 promotes the EMT process of Huh7 cells. ((a)-(c)) ARG1 impaired the expression of EMT-associated proteins at both protein (a, b) and mRNA (c) level, and overexpression of ARG1 significantly upregulated the expression of Vimentin, N-cadherin, and *β*-catenin, while ARG1 knockdown downregulated their expression. (d) ELISA assay suggests that ARG1 knockdown promotes the protein level of E-cadherin in Huh7 cells. N=3, ^*∗*^P<0.05 compared to the OE-NC; ^Δ^P<0.05 compared to the sh-NC.

Our data validated that ARG1 could promote the cell motility of HCC cells in vitro; further study was performed to explore the relevant mechanism. It is widely held that EMT constitutes a key process in cancer metastasis and progression. Therefore, the EMT-associated proteins were examined to evaluate the effect of ARG1 on EMT in HCC. As evident from Figures 5(a), 5(b), and 5(c), results of the Western blot and RT-PCR assays suggested that the protein and mRNA expression of mesenchymal markers Vimentin, N-cadherin, and *β*-catenin were significantly decreased in the shARG1 interference cells; meanwhile they were upregulated in ARG1 overexpressed cells. In addition, the level of E-cadherin protein, a key epithelial marker, was significantly increased in ARG1 knockdown cells; however, upregulation of ARG1 had no significant effect on the expression of E-cadherin (Figure 5(d)). As revealed by these results, ARG1 promotes the EMT process in HCC, leading to the promotion of ARG1 on the cell motility in HCC.

## 4. Discussion

It is widely held that the hallmarks of cancer include six biological capabilities as the results of genome instability and inflammation. Inflammation, which is a powerful component of the immune system, is one of the features of cancer and is involved in the occurrence and development of cancer [12–15]. Currently, numerous clinical and epidemiological investigations have highlighted that 15% to 20% of malignant tumors are the results of infections and uncontrolled inflammation. For instance, inflammatory bowel disease is associated with colon cancer, and chronic hepatitis B virus infection leads to liver cancer. With the development of researches, reprogramming of energy metabolism and evading immune destruction are also considered as important hallmarks of cancer [16]. Accordingly, some anti-inflammatory and immune-related genes have garnered extensive attention in the therapy of cancers.

Arginase is a pivotal metalloenzyme involved in hepatic urea cycle that metabolizes L-arginine to L-ornithine. Since L-ornithine and its metabolite—polyamine—are pivotal components involved in multiple fundamental cellular functions, including cell proliferation and cell membrane transport, arginase plays a key role in the cellular functions as well as various metabolic pathways [6].* ARG1* encodes the arginase I isoform, which is confirmed to be located in the cytoplasm and highly expressed in liver and M2 macrophages [9]. In addition to the metabolic enzyme activity in the hepatic urea cycle, ARG1 also constitutes a pivotal immune cell component. Previous studies have demonstrated that ARG1 is significantly involved in anti-inflammation, immune response, tumor immunity, and immunosuppression-related diseases for its metabolic enzyme activity in immune cells [6, 7, 17]. In an important aspect, recent studies have observed that ARG1 represents a sensitive and specific immunohistochemical marker for the hepatocellular differentiation [18–23]. Steggerda S M et al. report that ARG1 is positively expressed in the immune cells in a variety of tumors, in particular in NSCLC, gastrointestinal tract, and bladder; nevertheless, other than HCC, there is almost no expression in these tumor cells [24]. However, the role of ARG1 in the progression of HCC remains unclear.

In the current report, the immunohistochemistry assay was performed to assess the expression of ARG1 in HCC tissues and paracancerous tissues. We observed that ARG1 was substantially downregulated in HCC tissues in comparison with the corresponding paracarcinoma tissues. Besides, we also observed that the expression of ARG1 was associated with several clinicopathological features of HCC patients, that the higher expression of ARG1 was positively correlated with more aggressive tumor growth, size, ALT, and GGT level (Table 2), suggesting that ARG1 might function as an oncogene in the progression of HCC. In the most important manner, we figured out that the high expression of ARG1 had correlation with the poor DFS of HCC patients, indicating that ARG1 might be a prognostic biomarker for HCC patients. Moreover, the in vitro study revealed that ARG1 could enhance the cell viability, migration, and invasion of Huh7 cells, which further supported that ARG1 functions as an oncogene in HCC. Another arginase isoform—ARG2—is also revealed to be dysregulated in cancers and play different roles in different tissues and organs. Costa H et al. demonstrate that overexpression of ARG2 could promote proliferation, migration, and invasion in U-251 MG cells [25]. In clear cell renal cell carcinoma, reduced ARG2 activity promotes tumor growth through conserving the critical biosynthetic cofactor pyridoxal phosphate and avoiding toxic polyamine accumulation, indicating tumor-restricting properties of ARG2 [26]. Interestingly, the level of ARG2 was decreased in both ARG1 overexpressed and knockdown cells. Whether ARG2 is involved in the effect of ARG1 on tumor growth still requires further exploration in the future.

EMT, which is an essential process for the cell to gain the mesenchymal properties and motility, plays a pivotal role in tumor metastasis and progression [27]. N-cad and Vimentin are considered as pivotal mesenchymal markers, commonly used to reflect EMT [28]. In the cytoplasm, *β*-catenin continues binding and dissociating the cytoskeleton proteins, which promotes tumor cell migration by regulating cytoskeleton and modulating the coordinated cell–cell adhesion [29, 30]. As our data suggested, ARG1 could promote the EMT process in HCC cells through upregulating the expression of N-cad, Vimentin, and *β*-catenin both at protein and mRNA levels, which was further supported by the downregulation of N-cad, Vimentin, and *β*-catenin and upregulation of E-cadherin in ARG1 knockdown cells. As highlighted earlier, ARG1 promotes cell migration and invasion of HCC cells through enhancing the EMT process, indicating that targeting ARG1 might be a potential method to block HCC progression.

In summary, for the first time, we shed light on the fact that ARG1 is downregulated in HCC tumor and correlated with prognosis of HCC patients. We also demonstrate that ARG1 functions as an oncogene in the progression of HCC through promoting the EMT process. Our findings also provide a novel potential target for the therapy of HCC.

## Figures and Tables

**Table 1:** ARG1 expression in HCC compared with paracarcinoma tissue.

Group	n	ARG1 expression		P
		Low (n%)	High (n%)	
HCC	90	73 (81.1)	17 (18.9)	0.001_ _^*∗*^
para-carcinoma	90	12 (13.3)	78 (86.7)	

**Table 2:** ARG1 expression associated with the clinicopathological parameters in HCC.

clinicopathological parameters	n	ARG1 Low (n%)	ARG1 High (n%)	P
Gender				
Male	74	59 (79.7)	15 (20.3)	0.713
Female	16	14 (87.5)	2 (12.5%)	
Age (years)				
<60	69	54 (78.3)	15 (21.7)	0.350
≥60	21	19 (90.5)	2 (9.5)	
Tumor diameter (cm)				
<5	44	40 (90.9)	4 (9.1)	0.020_ _^*∗*^
≥5	46	33 (71.7)	13 (28.3)	
Tumor envelope				
Yes	47	40 (85.1)	7 (14.9)	0.311
No	43	33 (76.7)	10 (23.3)	
Relapse				
Yes	53	41 (77.4)	12 (22.6)	0.276
No	37	32 (86.5)	5 (13.5)	
Cirrhotic nodule				
Yes	78	64 (82.1)	14 (17.9)	0.853
No	12	9 (75.0)	3 (25.0)	
Pathological grading				
I-II	58	51(87.9)	7 (12.1)	0.026_ _^*∗*^
III-IV	32	22(68.8)	10 (31.2)	
DFS				
<12	21	13 (61.9)	8 (38.1)	0.027_ _^*∗*^
≥12	68	59 (86.8)	9 (13.2)	
TB				
<20*μ*mol/L	74	60 (81.1)	14 (18.9)	1.000
≥20*μ*mol/L	16	13 (81.3)	3 (18.7)	
ALT				
<40U/L	41	38 (92.7)	3 (7.3)	0.010_ _^*∗*^
≥40U/L	49	35 (71.4)	14 (28.6)	
GGT				
<40 U/L	24	24 (100.0)	0 (0)	0.014_ _^*∗*^
≥40U/L	66	49 (74.2)	17 (35.8)	
AFP				
<400ng/ml	54	48 (88.9)	6 (11.1)	0.106
≥400ng/ml	33	25 (75.8)	8 (24.2)	

## Data Availability

The data used to support the findings of this study are included within the article.
