# Corrigendum to “The Oncogenic Role of ARG1 in Progression and Metastasis of Hepatocellular Carcinoma”

**DOI:** 10.1155/2019/6212386

**Published:** 2019-12-13

**Authors:** Jia You, Wei Chen, Jing Chen, Qi Zheng, Jing Dong, Yueyong Zhu

**Affiliations:** Liver Research Center, First Affiliated Hospital of Fujian Medical University, Fuzhou 350005, Fujian Province, China

In the article titled “The Oncogenic Role of ARG1 in Progression and Metastasis of Hepatocellular Carcinoma,” [1] there was an error in the Acknowledgments section where the grant number 2016J0105 should be corrected to 2016J01529. The Acknowledgments section should be updated as follows:

This study was supported by the Natural Science Foundation of Fujian Province (No. 2016J01529).
